# Single-model deep learning approach for simultaneous cervical vertebral maturation staging and skeletal jaw relationship on lateral cephalograms using YOLOv8 and CNN

**DOI:** 10.1186/s12903-026-08578-y

**Published:** 2026-06-23

**Authors:** Naif Munawir Alotaibi, Ahmed Mohamed Kamel, Abeer Twakol Khalil, Shaza Mohammad Hammad

**Affiliations:** 1 Orthodontic Department, Faculty of Dentistry, Mansoura University, Mansoura, Egypt; 2 Department of Electronic and Communication Engineering, Faculty of Engineering, Mansoura University, Mansoura, Egypt

**Keywords:** Artificial intelligence, Cervical vertebral maturation, Skeletal classification.

## Abstract

**Background:**

This study aimed to develop an integrated artificial intelligence (AI) pipeline for cervical vertebral maturation (CVM) staging and skeletal jaw relationships and to validate its output against the results of human observers.

**Methods:**

A total of 720 lateral cephalograms were collected from the archives of the orthodontic department. The participants’ ages ranged from 8 to 18 years. Cephalograms were categorized into six cervical stages based on McNamara’s criteria and classified into skeletal Class I, II, and III patterns based on their ANB angles. The dataset was divided into a training set (*n* = 540) and a test set (*n* = 180). The training set was used to train a convolutional neural network (CNN) and a YOLOv8 model. The test set of cephalograms was coded and randomly assigned to two orthodontists for comparison with the AI model results using weighted kappa and Cohen’s kappa statistical analyses to verify accuracy.

**Results:**

For human observers, the interobserver agreement ranges were as follows: (κw ≈ 0.976–0.989) for skeletal classification and (κw ≈ 0.956–0.999) for CVM staging, while the intra-observer reliability was also almost perfect for both methods (κw ≈ 0.82– 0.85). Substantial agreement was found between the generated AI model and human observers for skeletal classification (κ = 0.725) and CVM staging (κ = 0.786). Both results were statistically significant (*p* < 0.01).

**Conclusions:**

Within the limitations of this study, the AI model exhibited substantial agreement with human observers for CVM staging and skeletal assessment, demonstrating its potential viability as a clinical decision-support tool.

## Background

Accurate assessment of skeletal maturation in orthodontics is essential for diagnosing growth status and optimizing treatment strategies, especially in cases requiring growth modification [1]. Various methods for assessing skeletal maturity rely on different growth indicators. A classical hand–wrist radiograph method involves observing the ossification stages of certain hand and wrist bones, which provides detailed information about the remaining growth potential [2]. The cervical vertebral maturation (CVM) method has gained popularity in present-day practice because it assesses morphological changes of the cervical vertebrae on routine lateral cephalograms without exposing patients to additional radiation [3]. According to prior studies, CVM and hand–wrist radiographs are among the most practical and reliable methods for treatment timing [2, 4].

Cephalometric radiography, a standard tool in orthodontics, is important for assessing craniofacial morphology, growth patterns, and treatment outcomes. Skeletal maturation is also possible using cephalometric analysis. The use of cervical vertebrae morphology to interpret skeletal maturation is referred to as the CVM method [2, 4]. Although advantageous, cephalometric radiographs have limitations. Two-dimensional representations of three-dimensional structures are subject to magnification errors, image distortions, and superimposition of bilateral structures, which may reduce accuracy [5]. Moreover, different head positioning and landmark identification can introduce examiner-dependent errors, thereby lowering the reproducibility [6]. Artificial intelligence (AI) is now being employed in orthodontics, offering the potential to overcome many of these limitations.

According to recent systematic reviews [7–9], AI applications in orthodontics are growing. One of its most common applications is to automate landmark identification on cephalometric radiographs [10, 11], assessment of skeletal maturation [12], and classification of craniofacial patterns [13]. Deep learning and machine learning models may enhance diagnostic accuracy while reducing inter-examiner variability and providing reproducibility across imaging modalities.

All these developments position AI as a powerful and transformative adjunct technology in orthodontics, with significant potential for skeletal maturation assessment and craniofacial classification.

Among deep learning approaches, YOLOv8 offers advantages for orthodontic applications due to its accurate and efficient landmark localization within a single unified framework, making it well-suited for cephalometric analysis. In parallel, convolutional neural networks (CNNs) remain highly effective for image-based classification tasks, such as cervical vertebral maturation staging, due to their capacity to learn hierarchical morphological features directly from radiographic data [14–16]. 

Although artificial intelligence has been extensively used to classify skeletal jaw relationship and CVM staging as separate applications, primary studies integrating both assessments within a single AI-based model remain scarce. Previous systematic reviews have examined these applications independently but have not reported on a combined approach [17, 18].

This study addresses this gap by testing the accuracy of an integrated artificial intelligence (AI) pipeline capable of assessing skeletal jaw relationship and CVM staging simultaneously. This study aimed to develop an AI model for cervical vertebral maturation (CVM) staging and skeletal jaw relationship assessment, and to validate its output against the results of human observers.

## Materials and methods

### Study design and ethical approval

This was an AI-model development and validation study using archived cephalograms, with model performance benchmarked against assessments by two human observers. The study was approved by the dental research ethics committee of the Faculty of Dentistry at Mansoura University in Egypt (code no. A0703024OR). The committee waived the need for signed informed consents.

### Collection and distribution of the dataset

Data were retrieved from the archival records of the Department of Orthodontics at Mansoura University, covering the period from 2020 to 2024. The study sample included male and female participants aged 8 to 18 years who were free of syndromic or craniofacial anomalies. High-quality digital radiographs without any artifacts, orthodontic appliances, or retainers were included. Low-quality and nonstandardized radiographs were excluded from the study.

The sample size was calculated using the PASS software (version 15, NCSS, Utah, USA). For deep learning analysis, a sample size of 540 cephalometric x-rays achieved 97% power to detect a true kappa value of 0.50 in a test of agreement between the human expert and AI using the kappa statistics. For kappa statistics, a sample size of 180 cephalometric x-rays achieved 80.9% power to detect a true kappa value of 0.50 in a test of agreement between the human expert and AI using a two-tailed kappa statistic. Both methods were based on a significance level of 0.050. Therefore, the total sample size was 720 images.

**Fig. 1:**
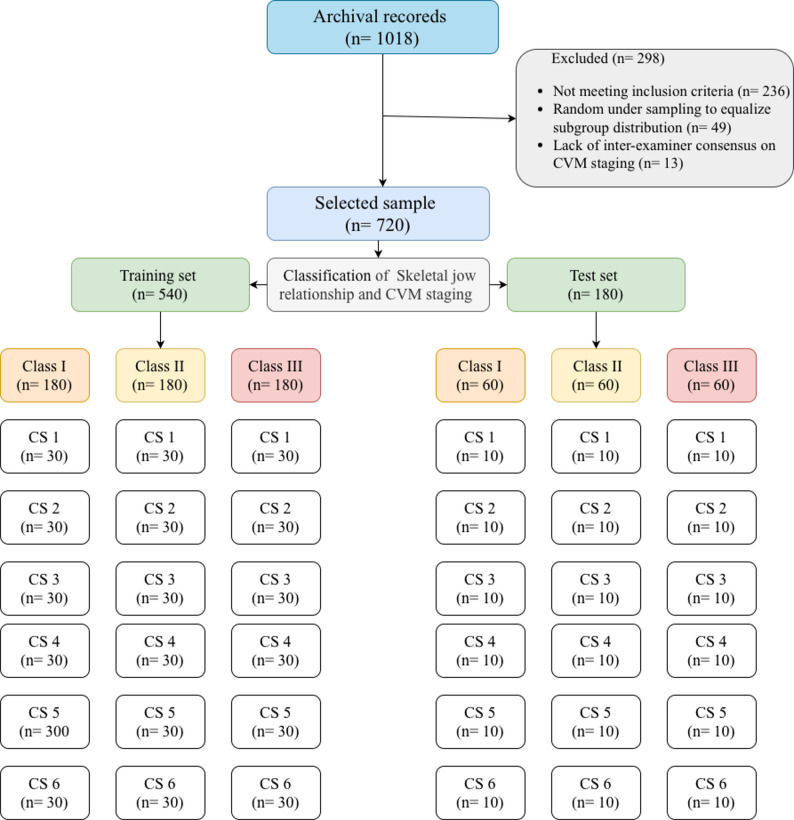
Sampling and random allocation of the radiographs within the study

A training set of 540 radiographs was used to develop the model, and another test set of 180 radiographs was used for evaluation of agreement between the AI model and two human observers (Fig. 1). There was no overlap between the two sets of data.

### Tracing and labeling

To optimize the accuracy of landmark detection and vertebrae classification, image processing was performed by AnyLabeling software (version 0.4.15, Neural Research Lab, Hanoi, Vietnam). This includes Contrast Limited Adaptive Histogram Equalization (CLAHE) to enhance local contrast, Gaussian blurring for noise reduction, and sharpening filters to emphasize the structural borders of the cervical vertebrae and cephalometric landmarks. All images of the dataset have been processed and saved in the training and test sets folders.

The YOLOv8l-Pose (Large) model was trained for 300 epochs with an image size of 1024 × 1024 and batch size of 16, utilizing the AdamW optimizer (lr0 = 0.001). A 5-fold cross-validation approach was employed to ensure result stability. For the MobilNetV2 classification network, hyperparameters were optimized using Random Search tuner across 10 trials. The model was trained with a batch size of 8 and an early stopping mechanism (patience = 10) to prevent overfitting and ensure optimal convergence.

**Fig. 2:**
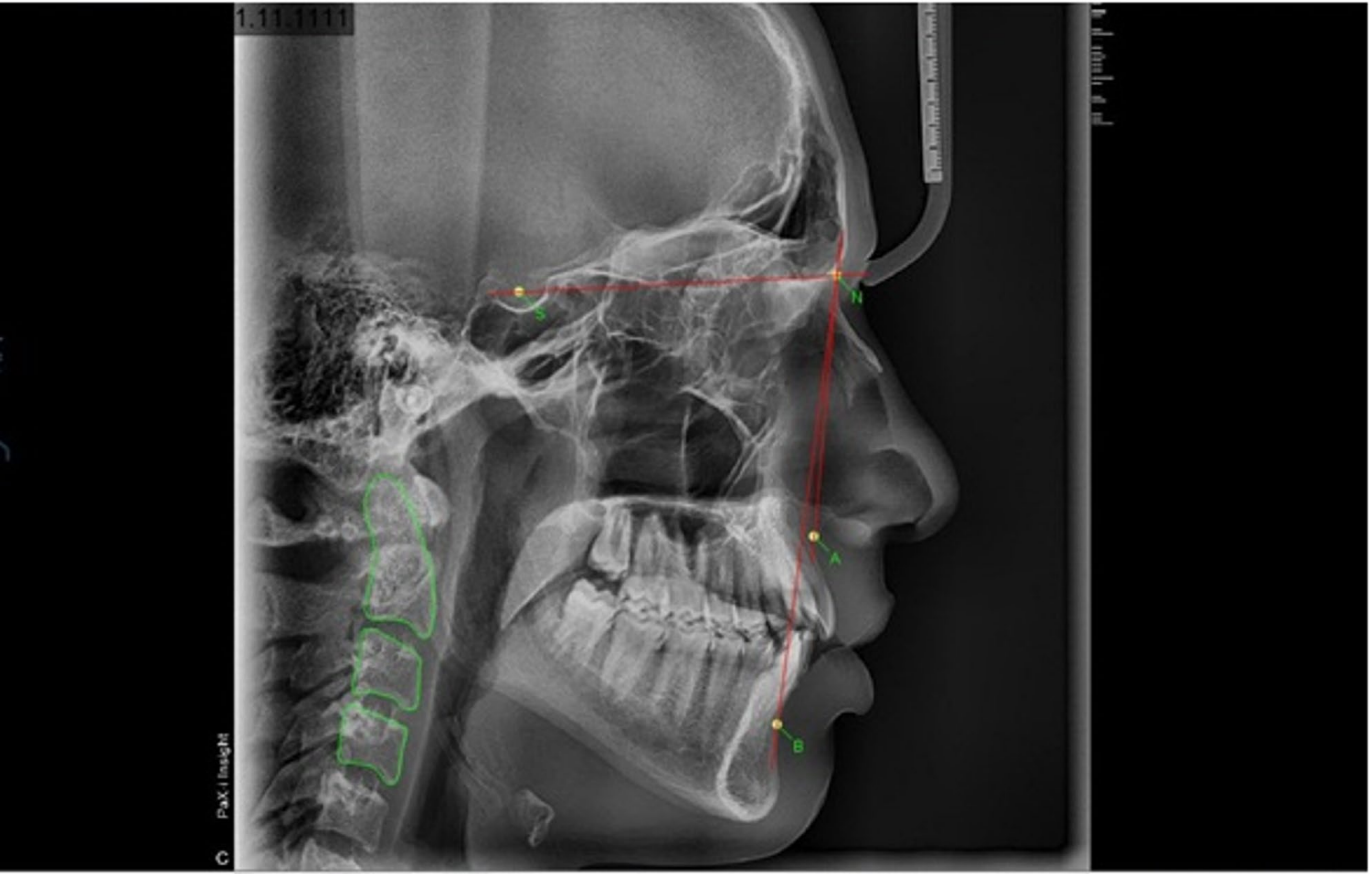
Cephalometric image represents the ANB angle and manual tracing for CVM

Lateral cephalograms (*n* = 720) were traced manually and independently by two experienced examiners using AudaxCeph software (version 6.1.4.3951, Audax d.o.o., Ljubljana, Slovenia). The radiographs were categorized into three groups (Class I, Class II, and Class III) according to the sagittal skeletal relationship defined by the ANB angle (Class I (0 < ANB < 4), Class II (ANB > 4), and Class III (ANB < 0) (Fig. 1). Subsequently, they were subdivided into six groups (CS 1, CS 2, CS 3, CS 4, CS 5, and CS 6) according to the CVM staging method proposed by McNamara et al. [19] (Fig. 2).

Radiographs demonstrating ambiguity in skeletal classification or CVM staging or those showing inter-examiner disagreement were excluded from the dataset.

After tracing and labeling, the images were divided into a training set (*n* = 540) and a test set (*n* = 180). The test set was kept strictly independent and was not involved in the training process.

### Model architecture

The dataset was trained using the deep learning model YOLOv8-Pose to assess sagittal skeletal relationships, which was adapted to automatically detect four cephalometric landmarks: Sella (S), Nasion (N), Point A (A), and Point B (B).

**Fig. 3:**
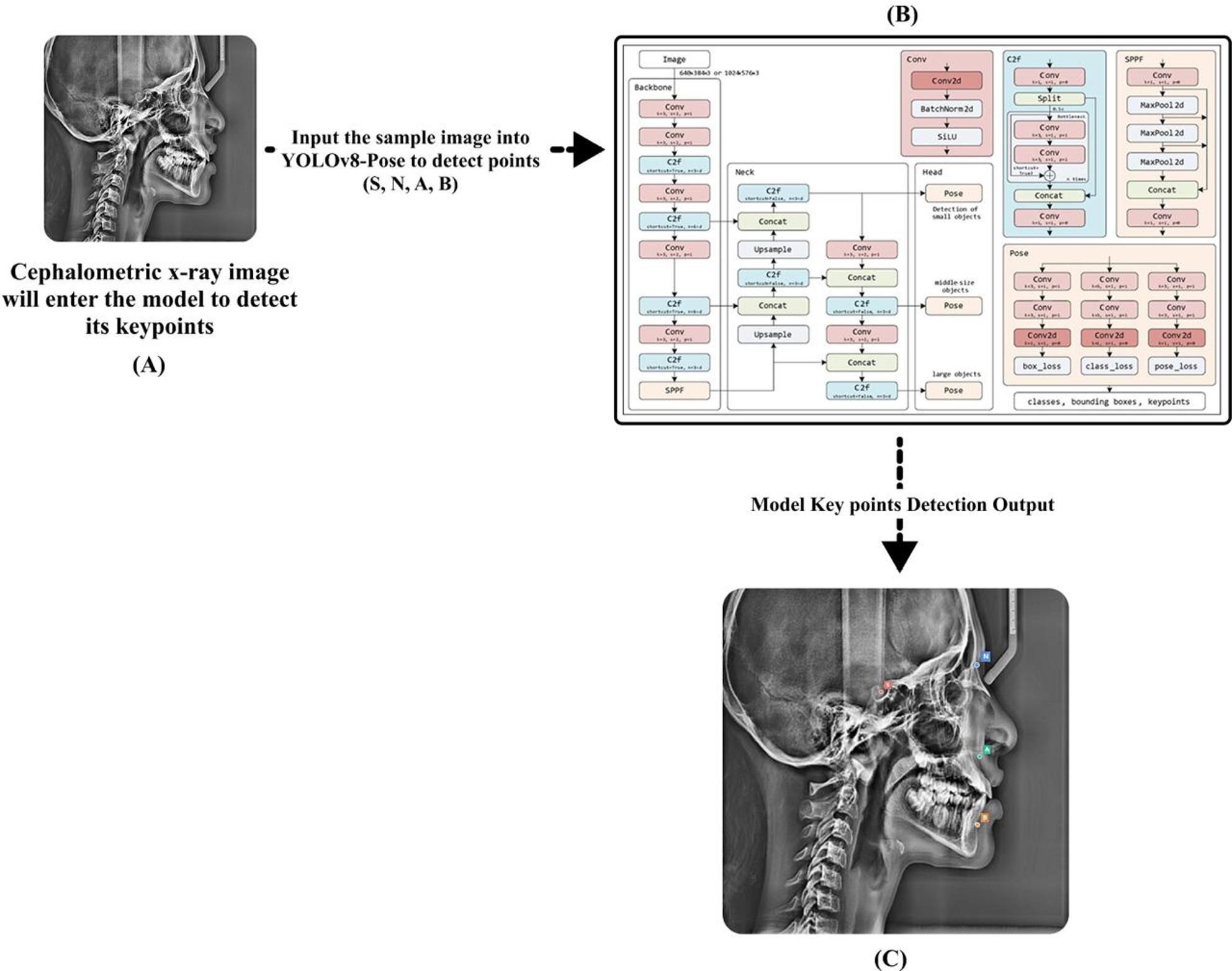
(**A**) shows a representative lateral cephalometric radiograph used as input to the model. **B** illustrates the architecture of the YOLOv8-Pose model [20] (**C**) displays the model’s output, where the detected cephalometric landmarks are superimposed on the original radiograph

The detected coordinates of landmarks S, N, A, and B were subsequently used to calculate the SNA and SNB angles. The ANB angle, derived from the difference between the SNA and SNB, was then used to classify the sagittal skeletal patterns as Class I, II, or III (Fig. 3).

**Fig. 4:**
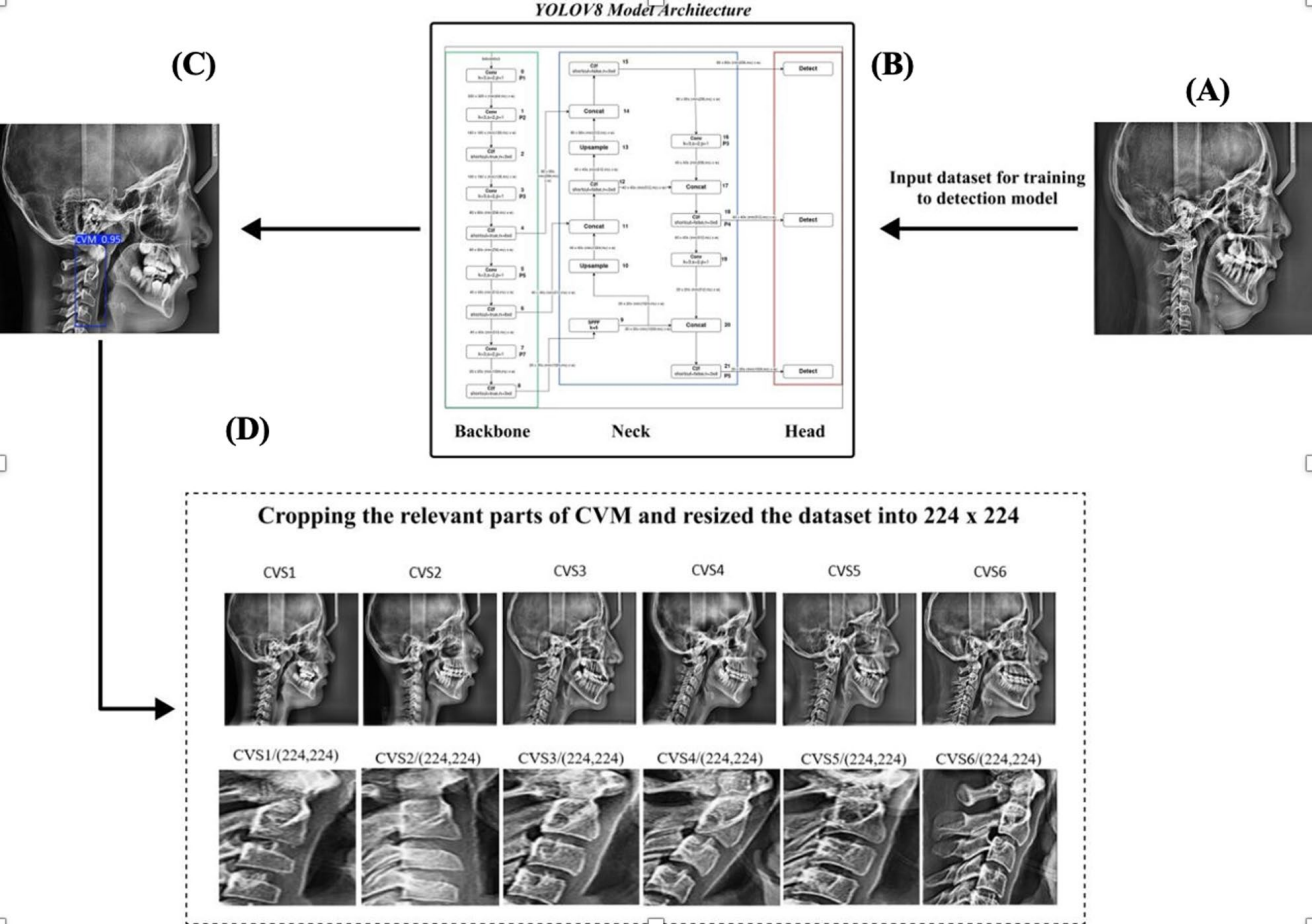
(**A**) A representative lateral cephalometric radiograph is shown as the input dataset used for training the detection model. **B** The YOLOv8 model architecture comprises backbone, neck, and head modules [21]. **C** The output of the trained model highlights the automatically detected cervical vertebral regions of interest overlaid on the original radiograph. **D** The relevant cervical vertebral regions (C2–C4) are cropped from the radiographs and uniformly resized to 224 × 224 pixels to create a standardized dataset for further analysis. The cropped images are subsequently classified into CVM stages (CS1–CS6)”

The CVM analysis was conducted in two stages. First, YOLOv8(Large) model was used to detect and localize the cervical vertebrae (C2–C4) from the cephalometric radiographs. After detection, the YOLOv8 model generated a bounding box encompassing the cervical vertebral region (C2–C4). This bounding box was used to automatically crop the region of interest from the original lateral cephalometric radiograph. The cropped cervical vertebral images were then resized to a fixed resolution of 224 × 224 pixels and normalized before being input into the CNN classifier. This procedure ensured that the classification network received standardized images focused exclusively on anatomically relevant structures, thereby minimizing background noise and inter-image variability. Features extracted using the McNamara method were assigned to a CVM stage by CNN (Fig. 4) [19]. 

This approach, featured in the detection–classification component, ensures that only the anatomically relevant vertebral regions are considered for maturation staging, thereby ensuring robust classification while minimizing computational overhead.

### Evaluation of the results – performance and statistical analysis

Cohen’s kappa and weighted kappa were analyzed in IBM SPSS Statistics (Version 27, IBM Corp, Armonk, NY, USA) to assess interobserver and intraobserver reliability. To evaluate inter-examiner reliability, 100 lateral cephalometric radiographs were randomly selected and read by the two examiners independently according to the evaluation criteria. For intra-examiner reliability, the time interval between two independent assessments of the same image was 3 weeks.

Several criteria were applied to assess the algorithm’s robustness. The classification model’s performance was evaluated quantitatively using accuracy, specificity, sensitivity, F1 score, and training and validation curves.

Another way to measure the network’s performance was through the confusion matrix, which shows the correctly and incorrectly predicted classes. The confusion matrix (often called the error matrix) is a specialized table used to visualize the algorithm’s performance and accuracy [22].

## Results

### Inter-observer reliability for skeletal jaw relationship and CVM

The interobserver reliability for skeletal jaw relationship demonstrated a perfect level of agreement, with a weighted kappa of 0.9838 (95% confidence interval: 0.976–0.989, *p* < 0.001). Similarly, CVM staging also showed perfect agreement between observers, with a weighted kappa of 0.977 (95% CI: 0.956–0.999, *p* < 0.001) (Table 1).

**Table 1:** Inter-observer reliability for skeletal jaw relationship measurement and CVM staging

Variable	Weighted kappa	95% CI	Sig.
skeletal jaw relationship	0.9838	0.976–0.989	< 0.001*
CVM staging	0.977	0.956–0.999	< 0.001*

*CVM* cervical vertebral maturation, *CI * confidence interval

*statistically significant at p ≤ 0.05, Weighted kappa

### Intra-observer reliability for skeletal jaw relationship and CVM staging

The intraobserver reliability analysis demonstrated excellent reproducibility for both skeletal jaw relationship and CVM staging (Table 2). For skeletal jaw relationship, the first and second examiners achieved weighted kappa coefficients of 0.850 (95% confidence interval [CI]: 0.842–0.864) and 0.843 (95% CI: 0.832–0.850), respectively, indicating almost perfect agreement (*p* < 0.001). Regarding CVM staging, the corresponding kappa values were 0.820 (95% CI: 0.800–0.814) and 0.832 (95% CI: 0.782–0.810), both of which were statistically significant at *p* < 0.001. These results reflect substantial to almost perfect consistency in the repeated evaluations.

**Table 2:** Intra-observer reliability for skeletal jaw relationship and CVM staging measurement

Variable	Weighted kappa	95% CI	Sig.
Intra-observer reliability for skeletal jaw relationship measurement			
Intra-examiner 1	0.85	0.842–0.864	< 0.001*
Intra-examiner 2	0.843	0.832–0.85	< 0.001*
Intra-observer reliability for CVM staging measurement			
Intra-examiner 1	0.82	0.80-0.814	< 0.001*
Intra-examiner 2	0.832	0.782–0.81	< 0.001*

*CVM* cervical vertebral maturation, *CI * confidence interval

*statistically significant at p ≤ 0.05, weighted kappa

### Training and validation performance of the AI models

**Fig. 5:**
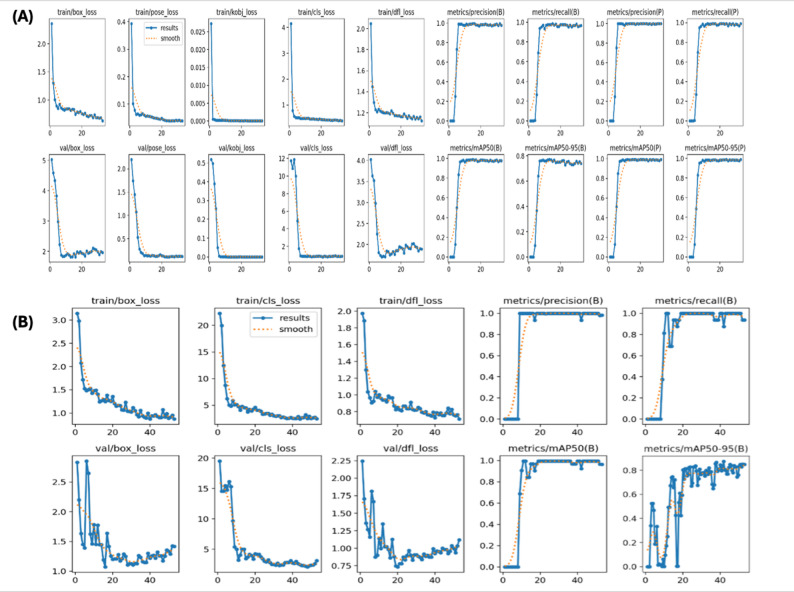
Training and validation performance graphs for (**A**) skeletal classification, (**B**) CVM detection

The training and validation curves demonstrated a consistent decline in box loss, pose loss, keypoint loss, classification loss, and distribution focal loss, indicating stable model learning without overfitting. The precision and recall values converged to 1.0 during training. For skeletal jaw relationship, the mean average precision (mAP) at an intersection over union (IoU) threshold of 0.5, mAP50 approached 1.0, with mAP50:95 reaching 85.4%. For CVM detection, mAP50 also approached 1.0, while mAP50:95 reached 82.7%. These results confirm the high detection accuracy and robust localization across varying overlap thresholds (Fig. 5).

### Model classification performance regarding skeletal malocclusion and CVM stages

Confusion matrices for skeletal jaw relationship and CVM stage predictions were generated using the test set to visualize the model’s performance. In both tasks, most predictions were aligned along the diagonal line, indicating reliable classification, whereas off-diagonal entries represented misclassifications.

**Fig. 6:**
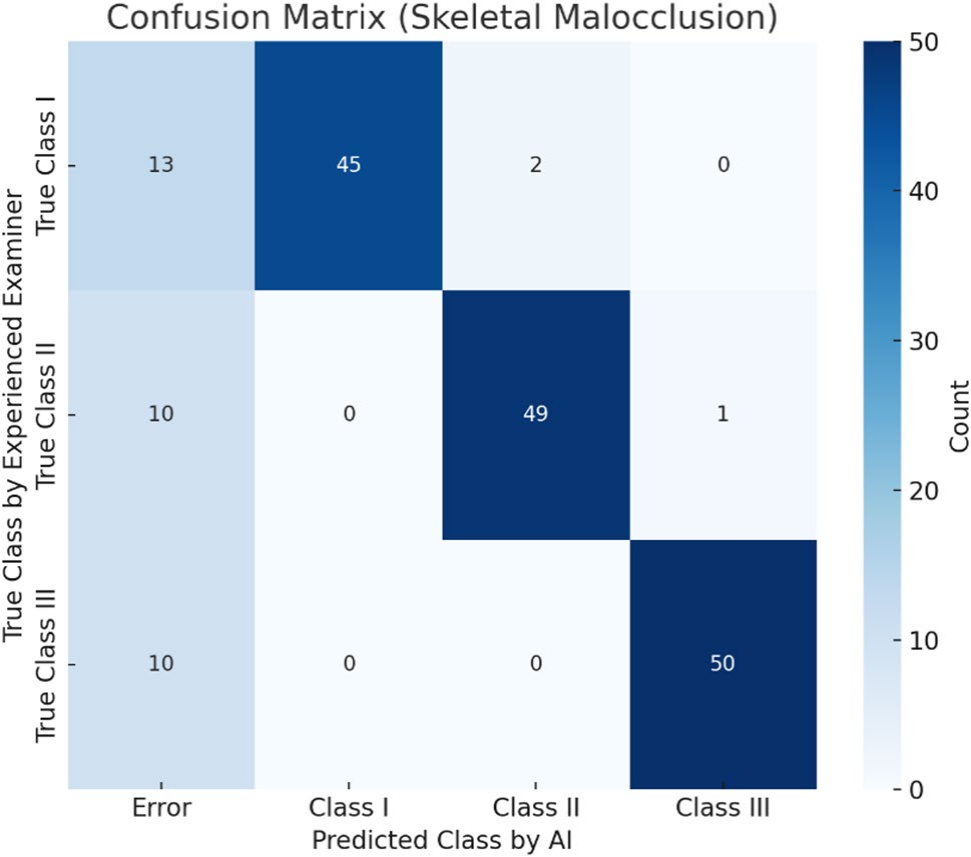
Confusion matrix for skeletal jaw relationship predictions obtained by the model on the test set

**Fig. 7:**
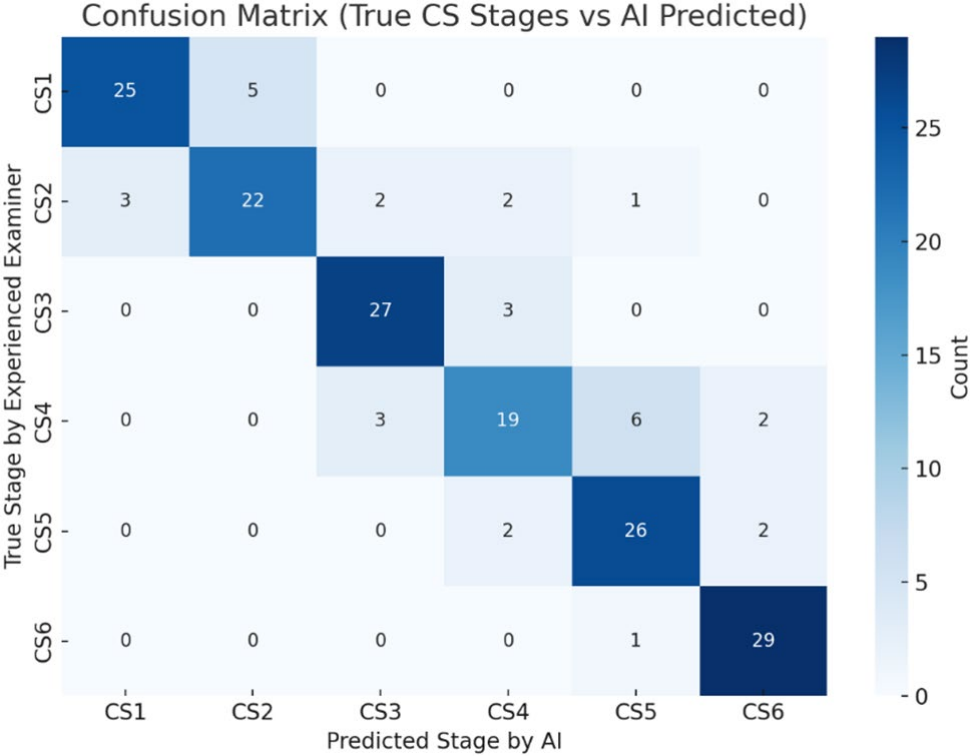
Confusion matrix for the CVM stage predictions obtained by the model on the test set

Precision, accuracy, recall, and F1-scores were calculated from the corresponding confusion matrices for further assessment of the model classification performance regarding skeletal jaw relationship and CVM stages (Figs. 6 and 7), with the results presented in Tables 3 and 4.

**Table 3:** Precision, recall, F1-scores, and Accuracy of the model on the test set for the skeletal jaw relationship classes

Classes	Precision	Precision (95% CI)	Recall	Recall (95% CI)	F1-score	F1-score (95% CI)	Sample size Of each group
Class I	1.00	0.94-1.00	0.75	0.64–0.86	0.86	0.77–0.95	60
Class II	0.96	0.91-1.00	0.82	0.72–0.92	0.88	0.80–0.96	60
Class III	0.98	0.94-1.00	0.83	0.73–0.93	0.90	0.83–0.97	60
Accuracy	85.4%						180

Precision = TP / (TP + FP); Recall = TP / (TP + FN); F1-score = 2 * Precision * Recall / (Precision + Recall); Accuracy = (TP + TN) / (TP + TN + FP + FN) TP is true skeletal malocclusion classifications, FP is false positive, FN is false negative, and TN is true negative. Precision, Recall, F1-score, and Accuracy were calculated from the confusion matrix shown in Fig. 5

**Table 4:** Precision, recall, F1-scores, and Accuracy of the model on the test set for the CVM staging

CVM Stage	Precision	Precision (95% CI)	Recall	Recall (95% CI)	F1-Score	F1-score (95% CI)	Sample size Of each subgroup
CS 1	0.86	0.74–0.98	0.83	0.70–0.96	0.85	0.72–0.98	30
CS 2	0.79	0.64–0.94	0.73	0.57–0.89	0.76	0.61–0.91	30
CS 3	0.79	0.64–0.94	0.90	0.79-1.00	0.84	0.71–0.97	30
CS 4	0.73	0.57–0.89	0.63	0.46–0.80	0.68	0.51–0.85	30
CS 5	0.76	0.61–0.91	0.87	0.75–0.99	0.81	0.67–0.95	30
CS 6	0.88	0.76-1.00	0.97	0.91-1.00	0.92	0.82-1.00	30
Accuracy					82.7%		180

Precision = TP / (TP + FP); Recall = TP / (TP + FN); F1-score = 2 * Precision * Recall / (Precision + Recall); Accuracy = (TP + TN) / (TP + TN + FP + FN) TP is true CS, FP is false positive, FN is false negative, and TN is true negative. Precision, Recall, F1-score, and Accuracy were calculated from the confusion matrix shown in Fig. 6

The model was evaluated on 180 images distributed across three skeletal classes and the 6 CVM subgroups. The skeletal jaw relationship accuracies were 75% for Class I, 81.7% for Class II, and 83.3% for Class III. The agreement between the AI system and the two experienced examiners was assessed using Cohen’s kappa coefficient. Concordant classifications were obtained for 45 Class I, 49 Class II, and 50 Class III radiographs, with errors and cross-class assignments exhibiting discrepancies (Table 5). Despite these inconsistencies, the overall agreement was substantial (κ = 0.725, *p* < 0.01).

**Table 5:** Comparison of skeletal malocclusion classifications by AI system and the 2 experienced examiners

			Skeletal jaw relationship by the 2 experienced examiners			Total
			CI	CII	CIII	
Skeletal jaw relationship by AI	0	Count	13	10	10	33
		% within Class by the 2 experienced examiners	21.7%	16.7%	16.7%	18.3%
	CI	Count	**45**	0	0	45
		% within Class by the 2 experienced examiners	75%	0.0%	0.0%	25%
	CII	Count	2	**49**	0	51
		% within Class by the 2 experienced examiners	3.3%	81.7%	0%	28.3%
	CIII	Count	0	1	**50**	51
		% within Class by the 2 experienced examiners	0.0%	1.7%	83.3%	28.3%
Total		Count	60	60	60	180
		% within Class by the 2 experienced examiners	100%	100%	100%	100%

*0* error, *CI * skeletal class I, *CII* skeletal class II, *CIII* skeletal class III, *AI* artificial intelligence, *%* percentage

bold numbers indicate correct scores between the AI system and the examiner

The CVM stage prediction achieved accuracy rates of 83.3%, 73.3%, 90.0%, 63.3%, 86.7%, and 96.7% for CS1, CS2, CS3, CS4, CS5, and 96.7% for CS6. Identical classifications were obtained for 25 of 30 radiographs at CS1, 22 at CS2, 27 at CS 3, 19 at CS4, 26 at CS5, and 29 at CS6. The agreement between the AI system and the experienced examiner in classifying 180 radiographs across the six stages was substantial, with a weighted kappa value of 0.786 (*p* < 0.01) (Table 6).

**Table 6:** Comparison of CVM staging classifications by AI system and the 2 experienced examiners

			CVM staging by the 2 experienced examiners						Total
			CS1	CS2	CS3	CS4	CS5	CS6	
CVM staging by AI	CS1	Count	**25**	5	0	0	0	0	30
		% within CVM by the 2 experienced examiners	83.3%	16.7%	0%	0%	0%	0%	16.7%
	CS2	Count	3	**22**	2	2	1	0	30
		% within CVM by the 2 experienced examiners	10.0%	73.3%	6.7%	6.7%	3.3%	0.0%	16.7%
	CS3	Count	0	0	**27**	3	0	0	30
		% within CVM by the 2 experienced examiners	0%	0%	90%	10%	0%	0%	16.7%
	CS4	Count	0	0	3	**19**	6	2	30
		% within CVM by the 2 experienced examiners	0%	0%	10%	63.3%	20%	6.7%	16.7%
	CS5	Count	0	0	0	2	**26**	2	30
		% within CVM by the 2 experienced examiners	0%	0%	0%	6.7%	86.7%	6.7%	16.7%
	CS6	Count	0	0	0	0	1	**29**	30
		% within CVM by the 2 experienced examiners	0%	0%	0%	0%	3.3%	96.7%	16.7%
Total		Count	28	27	32	26	34	33	180
		% within CVM by the 2 experienced examiners	16%	15%	18%	14%	19%	18%	100.0%

*AI* artificial intelligence, *CVM* cervical vertebral maturation, *CS* cervical stage, *% * percentage

bold numbers indicate correct scores between the AI system and the examiner

**Fig. 8:**
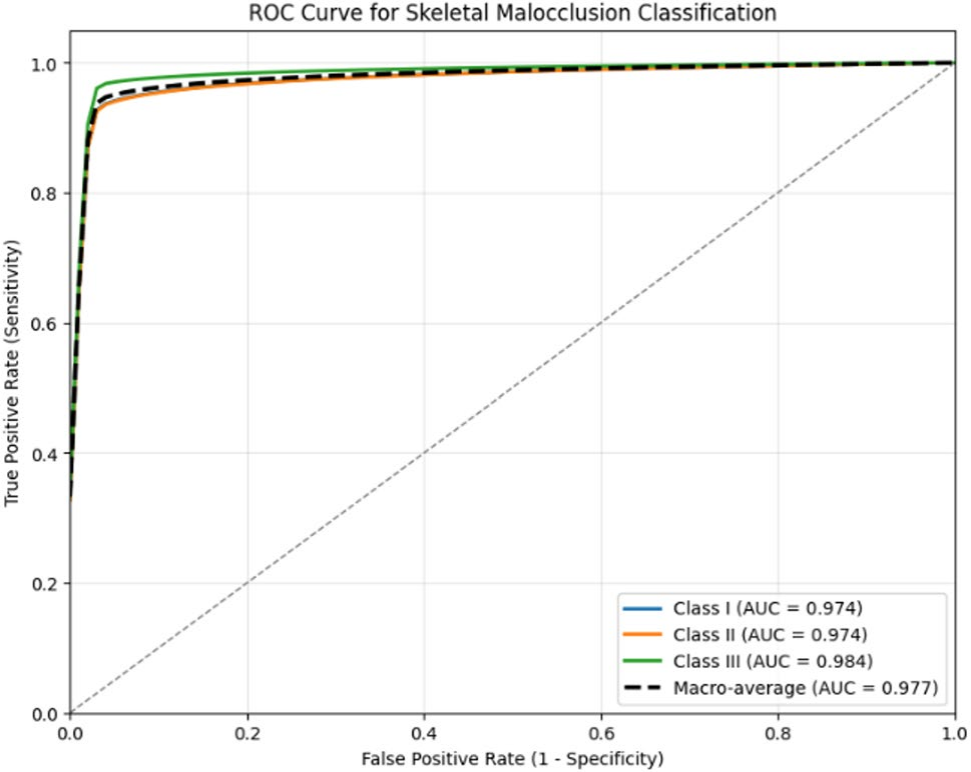
SimplePara>ROC curves for skeletal jaw relationship classification. AUC values demonstrate high diagnostic performance for all classes

**Fig. 9:**
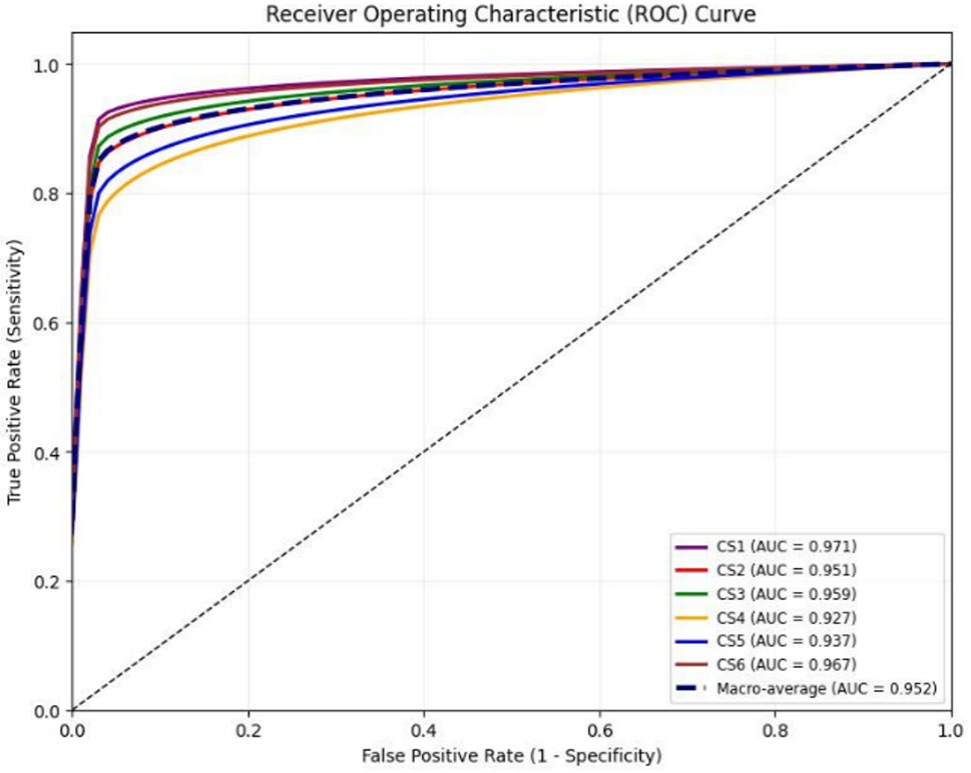
ROC curves for CVM staging classification. Receiver operating characteristic (ROC) curves are presented for each class (CS1–CS6) and the macro-average. The area under the curve (AUC) indicates high discriminative performance of the proposed model

The classification efficiency was analyzed using Receiver Operating Characteristic (ROC) curves on the test dataset. For skeletal jaw relationship classification in (Fig. 8**)**, the AUC values of 0.974, 0.974, and 0.984 for Classes I, II, III resulted macro-average AUC of 0.977. The CVM staging model achieved high diagnostic accuracy across all maturation phases with AUC values ranging from 0.927 in CS4 to 0.971 in CS1 and macro-average AUC of 0.952 in (Fig. 9).

It’s noteworthy that while these AUC values indicate near perfect classification, the inherent nature of multiclass ROC analysis should be considered. The plotting of these curves requires transforming the multiclass problem into multiple binary tasks (One-vs-Rest). This mathematical transformation creates a temporary state of class imbalance, where the single positive class is tested against the aggregate of all other negative classes. This can cause higher AUC values than those observed in other metrics. To ensure a conservative and clinically realistic assessment, these results were validated with macro-averaged F1 scores and 95% Confidence Intervals (CI), providing a more rigorous evaluation of models’ reliability on unseen data.

## Discussion

Because of the significant variation in growth patterns among individuals, an accurate assessment of an individual’s skeletal classification and growth status is essential for effective diagnosis and treatment planning.

The general CNN architectures and the specialized YOLOv8 model, which were used in the current study, are revolutionizing orthodontics by automating and improving diagnostic image analysis. YOLOv8 is fast and accurate, capable of identifying and segmenting objects. Therefore, it is useful for the initial detection and localization of anatomical structures and pathologies before performing further classification and detailed analysis with other more specialized CNN models [23, 24]. Therefore, CNN and YOLOv8 were deemed the most appropriate choices for our study’s goals.

The use of artificial intelligence (AI) for skeletal jaw relationship is a burgeoning research area in orthodontics. This study analyzed the ability of an AI system to analyze skeletal relationships using SNA, SNB, and ANB values and first assessed how closely the AI classifications were aligned with those of two experienced examiners. The findings showed substantial agreement between the AI model and the examiners (Cohen’s kappa coefficient: 0.725, *p* < 0.01). According to Paddenberg-Schubert et al. [25], AI can effectively determine skeletal classes, with a satisfactory kappa value of 0.73, which is consistent with the present study.

Kim et al. [26] used CNNs and reported an accuracy of 96% against an AI-based tracing software. The current study’s accuracy is 85.4%. This discrepancy could be attributed to the larger dataset in Kim’s study (1,574 images, including 1,334 for training), which likely enhanced the model’s performance.

Jayathilake et al. [27] reported accuracies for malocclusion prediction of 88.89%, 83.33%, 88.89%, and 55.56% for the multinomial logistic regression model, k-nearest neighbor (K-NN) method, random forest, and Naïve Bayes, respectively. The present study produced an accuracy of 85.4% using a CNN with the YOLOv8 algorithm. This difference may reflect the distinct classification mechanisms and inherent characteristics of the applied machine learning models, which contribute to variations in the predictive performance.

The AI model achieved accuracy rates of 75% for Class I, 81.7% for Class II, and 83.3% for Class III. The skeletal classes form a continuous spectrum, indicating that Class I lies between classes II and III. This means that the Class I skeletal pattern beyond the minimum and maximum range shares features with mild Class II and Class III malocclusion. Thus, it is difficult to classify it. In addition, the ANB angle can change independently of jaw position in the anteroposterior direction because of variation in maxillary and mandibular dimensions, dental position, and other factors [28, 29]. According to Li et al. [13], the sagittal skeletal patterns were classified with greater accuracy in Class III, followed by Classes II and I, when using CNNs. The same trend was observed in the present study. This may be because Class I cases are more common and lack distinctive visual features compared to the other two classes, allowing CNNs to reliably capture typical morphological features.

This study also showed that ML models trained on highly consistent datasets can improve the accuracy and reliability of CVM staging, thereby minimizing the subjective bias involved in the manual evaluation.

The interobserver reliability in the dataset produced a Cohen’s kappa of 0.97, which is in agreement with the agreement between the two examiners. This finding reaffirms that the CVM method is a robust tool for assessing a patient’s growth stage. Remarkably, this level of agreement is much higher than that reported by Nestman et al. [30] (0.62), Rainey et al. [31] (0.70), Morris et al. [32] (0.88), and Rongo et al. [33] (0.78). Conversely, Santiago et al. [34] reported that the CVM method is insufficiently reliable and valid, highlighting the difficulty of achieving consistent assessments. Variability is often linked to the subjective nature of visual assessment and variability in the experience of the examiner [35, 36].

In the present study, the AI model developed to predict the CVM stage based on 540 lateral cephalometric radiographs showed an accuracy of 82.77%, with a Cohen’s kappa value of 0.786.

Several previous studies have reported comparable or higher levels of agreement between AI models and human examiners in the classification of CVMs. Baptista et al. [37], Padalino et al. [38], and Dzemidzic et al. [39] reported Cohen’s kappa values of 0.861, 0.94, and 0.985, respectively. These higher values may be attributed to smaller datasets (188, 100, and 99 images, respectively) and different models (Naïve Bayes algorithm, delta-dent, and Cephalometar HF V1). Differences in methodological approaches may also have contributed. Dzemidzic et al. [39] used the criteria established by Hassel and Farman (2015), whereas the present study applied a different method [19]. Santiago et al. [40] reported a comparable accuracy of 83.2% from a smaller dataset of 236 samples; thus, the similarity may be due to overfitting rather than generalizability.

On the other hand, Kim et al. [12] used a different AI architecture on a dataset of the same size (with 720 samples) but achieved a markedly lower accuracy of 62.5%, likely due to variations in network design and training strategy. Amasya et al. [41] designed a study using four observers and an ANN model, where the ANN model demonstrated low agreement with the observers (κ = 0.40–0.55), likely due to the limited dataset of 72 radiographs visually evaluated from a total of 647 available images. These comparisons highlight that both dataset size and model architecture are critical determinants of AI performance and must be carefully optimized to ensure robust and generalizable CVM stage prediction outcomes.

Tentaş and Özden [42] employed separate YOLOv8x models to evaluate multiple hand–wrist maturation systems, whereas the present study adopts a unified YOLOv8–CNN pipeline applied to lateral cephalograms, enabling simultaneous assessment of cervical vertebral maturation using the McNamara method [19]. In the present study, CVM stage classification achieved F1-scores ranging from 0.68 to 0.92, in comparison, Tentaş and Özden reported higher F1-scores (0.92–0.99), which likely reflects differences in imaging modality, methodological design, and model architecture, as hand–wrist maturation stages are generally more morphologically distinct, whereas cervical vertebral maturation exhibits greater anatomical overlap, particularly at transitional stages [1, 43]. 

Despite the promising findings of this study, the following limitations should be considered. The single-center retrospective design and relatively small sample size may limit the generalizability to external populations, necessitating multi-center validation. Furthermore, the inherent limitations of two-dimensional cephalograms, including magnification, distortion, and structural superimposition, may limit assessment accuracy compared to three-dimensional modalities such as cone-beam computed tomography. Most importantly, radiographs with ambiguous findings or inter-examiner disagreement were excluded, which likely inflates kappa values and model performance relative to real-world clinical conditions, where borderline and transitional stages represent the primary diagnostic challenge. Future multi-center prospective studies incorporating ambiguous cases, large and diverse datasets, and three-dimensional imaging integration are essential to translate these findings into routine clinical practice.

## Conclusion

1. Interobserver agreement was excellent for both skeletal classification (κw = 0.976–0.989) and CVM staging (κw = 0.956–0.999), with almost perfect intraobserver reliability (κw ≈ 0.82–0.85), confirming the consistency of human observer assessments within this dataset.
2. AI model achieved substantial agreement with human observers for skeletal classification (κ = 0.725) and CVM staging (κ = 0.786), with statistical significance.
3. While the AI-based algorithms demonstrated feasibility as a promising integrated pipeline for assessing CVM stages and skeletal relationships, the findings are limited by the single-center data and the exclusion of ambiguous cases. Routine clinical implementation requires further external validation to improve sensitivity around transitional stages.

## Data Availability

All datasets used and analyzed during the current study are available from the corresponding author on reasonable request.

## Abbreviations

AI: Artificial intelligence
CNN: Convolutional neural network
CVM: Cervical vertebral maturation
YOLOv8: You Only Look Once Version 8
IoU: Intersection over Union
mAP: Mean Average Precision
mAP50: Mean Average Precision at 0.50 IoU threshold
mAP95: Mean Average Precision at 0.50 to 0.95 IoU thresholds
TP: True positive
TN: True negative
FP: False positive
FN: False negative
KW: Weighted kappa
K: Kappa
